# Effects of the online computerized cognitive training program BEYNEX on the cognitive tests of individuals with subjective cognitive impairment and Alzheimer’s disease on rivastigmine therapy

**DOI:** 10.3906/sag-1905-244

**Published:** 2020-02-13

**Authors:** Nilgün ÇINAR, Turker A.H. SAHINER

**Affiliations:** 1 Department of Neurology, Faculty of Medicine, Maltepe University, İstanbul Turkey; 2 Department of Neurology, Memorial Şişli Hospital, İstanbul Turkey

**Keywords:** Computerized cognitive training, Alzheimer’s disease, subjective cognitive impairment, rivastigmine

## Abstract

**Background/aim:**

Clinical trials conducted on the efficacy of computerized cognitive training (CCT) programs have not led to any important breakthroughs. CCT is a safe and inexpensive approach, but its efficacy in patients on rivastigmine therapy has not been evaluated. This study aims to compare effects of CCT and examines rivastigmine to determine whether CCT has any further contributions to make.

**Materials and methods:**

Sixty individuals with subjective memory complaint (SCI) and 60 individuals with early stage Alzheimer’s dementia (AD) were subjected to the Montreal Cognitive Assessment (MoCA), Cambridge Cognition (CANTAB tests: MOT, PRM, DMS, SWM, PAL, RTI), and Bayer-ADL. After screening patients who were diagnosed with AD, we started rivastigmine patch treatment (10 cm2 = 9.5 mg). The SCI and AD groups were randomly divided, and one each of the SCI and AD groups were accessed using BEYNEX, a web-based program. After a minimum of at least 1200 min of use, the diagnostic tests were repeated.

**Results:**

The AD groups’ MoCA scores of the BEYNEX-practicing group demonstrated meaningfully increase, whereas they decreased in the control group, and the Bayer-ADL scores indicated improvement in ADL. The CANTAB tests both in SCI and AD and in groups using BEYNEX showed positive improvement in MOT, DMS, and PAL data.

**Conclusion:**

This study is a rare example that focuses on both groups with SCI and AD. The efficacy of CCT varies across cognitive domains and shows significant efficacy for AD but small improvements in cognitively healthy older adults. In future studies, integration with a smart learning algorithm may lead to interesting observations on which parameters are more sensitive to change under long-term use of CCT in a large number of subjects.

## 1. Introduction

Computer cognitive training (CCT) programs for elderly people are a relatively new and developing option for cognitive rehabilitation and continue to be adopted in many fields of research. CCT is implemented through group sessions and individual training using a computer-based program modifying protocols previously shown to be effective in randomized controlled trials [1]. Clinical trials conducted on the efficacy of CCT have not led to any important breakthroughs, yet there is a growing consensus that this can, at least partially, be explained by methodological difficulties. Although adoption is slow in clinical research, change is inevitable. Cognitive decline and memory impairment are a difficult and expensive aspect of aging [2]. Age-related cognitive impairment rates affect about 15%–25% of the elderly population, which makes it nearly twice as common compared to dementia [3–5]. The health-related costs are nearly 44% higher for elderly patients with mild cognitive impairment compared to those who do not have any impairment.

Considering cognitive decline and impairment as the essential criteria of dementia, it is important to underline the significant economic and health-related costs of the caring process and the attempts to prevent or slow down the decline [6]. There are numerous studies demonstrating the positive effects of acetylcholinesterase (AChE) inhibitors on cognitive tests in the first 6 months. All AChE inhibitors have shown greater efficacy than placebos in randomized, double-blind, parallel-group clinical trials [7]. This study observed the effects of CCT on patients on rivastigmine, which is also an AChE inhibitor. It also compared the effects on Alzheimer’s dementia (AD) patients with a subjective memory complaint (SCI) group, comprising patients not on any medication. CCT is a safe and inexpensive approach, but its efficacy in patients on rivastigmine therapy has not yet been evaluated. This study aims to compare the effects of CCT and examines rivastigmine to determine whether CCT has any further contributions to this effect.

## 2. Materials and methods 

This study was conducted between 1 January and 31 December 2017 at five study sites in İstanbul, Turkey. Three study sites did not complete the study. Subjects were recruited in the memory clinics of different hospitals. Out of 141 individuals aged between 50 and 85 screened for eligibility, 120 subjects were enrolled in the study. 

Exclusion criteria included a history of severe psychiatric or neurologic disorders, a moderate stage of dementia changes with antidementive or antidepressive medication within 3 months prior to study initiation, or physical conditions that would prevent participation in the physical training program. The level of education of subjects was at least secondary school. Depression was excluded with the Geriatric Depression Scale (GDS), comprising 30 items, developed by Yesavage to evaluate depression [8]. Sixty individuals with SCI [9] and 60 individuals with probable AD, as defined by the National Institute of Neurological and Communicative Disorders and Stroke/Alzheimer’s Disease and Related Disorders Association (NINCDS-ADRDA) criteria [10], were subjected to the Montreal Cognitive Assessment (MoCA) [11] and Cambridge Cognition CANTAB cognitive assessment software. The CANTAB tests included motor screening (MOT), pattern recognition memory (PRM), delayed matching sample (DMS), spatial-working memory (SWM), paired associated learning (PAL), reaction time (RTI) [12], and Bayer-ADL tests [13]. We accepted SCI patients as individuals who reported a worsening of their thinking abilities, including memory, but for whom the decline could not be verified by standard tests [9]. 

**Figure 1:**
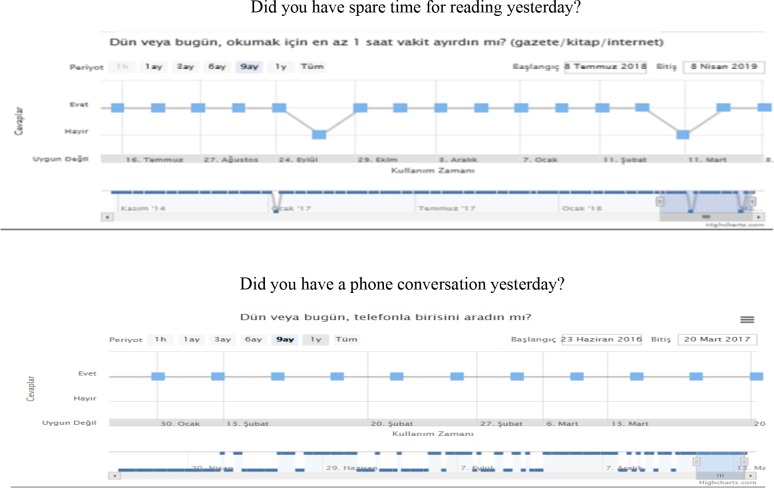
Questions of daily living activities (2 examples of 25 questions).

**Figure 2:**
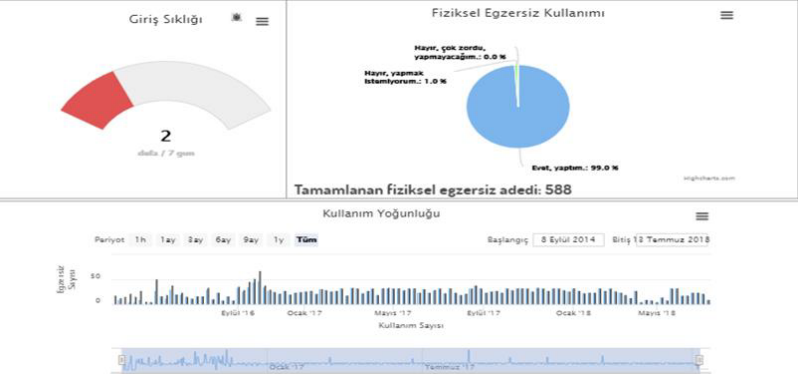
Patient total performance screen.

**Figure 3:**
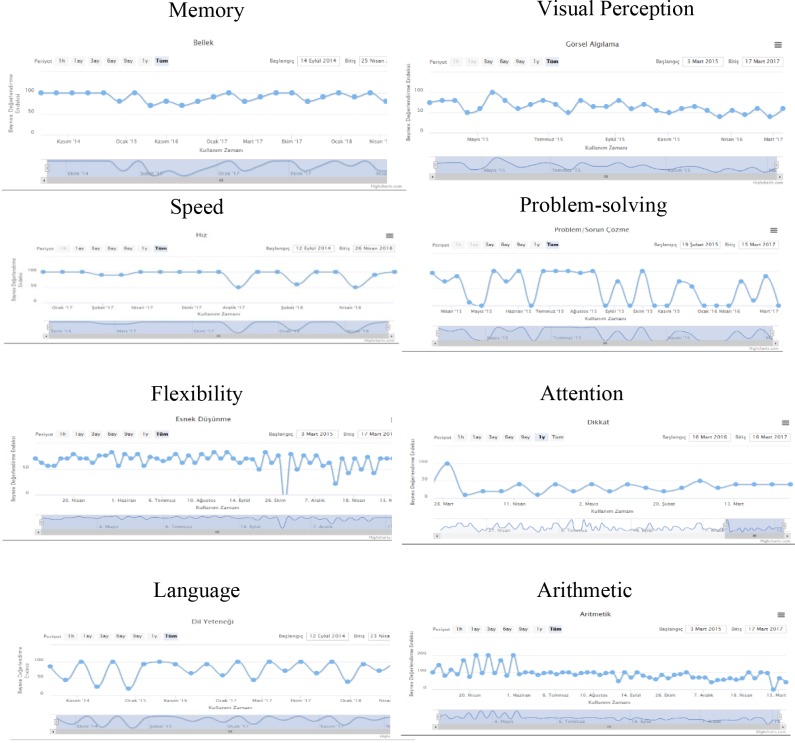
Patient performance screen for 8 different parameters (Memory, Visual Perception, Speed, Problem-Solving, Flexibility, Attention, Language Skills, Arithmetic).

After screening patients who were diagnosed with AD, rivastigmine patch treatment (10 cm2 = 9.5 mg) was started. Later, the SCI and AD groups’ age and education levels were normalized and divided into two subgroups consisting of 30 subjects. The SCI and AD subgroups were provided a password to access the web-based BEYNEX program and were asked to complete tasks, which included playing 3 different 5-min-long computer games, practicing a 3-min-long physical exercise video on a daily basis, and answering questions on Bayer-ADL once a week (Figure 1). All tasks were designed within the parameters used by clinicians and took 15–20 min to complete daily. The BEYNEX exercises are completely unique and are not in commercial use, and the initial research outcomes were presented at the Alzheimer’s Association International Conference (AAIC 2018/Technology and Dementia, Chicago, IL, USA) [14]. Patients’ total activity time (Figure 2), Bayer-ADL scores, and game performances were monitored only by clinicians using 9 different parameters (ADL, Memory, Visual Perception, Speed, Problem-Solving, Flexibility, Attention, Language Skills, Arithmetic) (Figure 3). After 3 months or at least 1200 min of BEYNEX use, the diagnostic tests (MoCA, CANTAB, B-ADL) were repeated for both test and control groups. 

SPSS 16.0 (SPSS Inc., Chicago, IL, USA) was used for statistical analyses. Statistical analysis was performed using one-way ANOVA test with post hoc analysis using the Tukey test.

## 3. Results

All subjects’ routine laboratory test results were within normal limits, including complete blood cell count, serum electrolyte levels including magnesium and glucose, serum chemistry panel (BUN/creatinine), liver function tests, thyroid function tests, and serum vitamin B12 tests. One hundred and forty-one participants were enrolled in the intervention study. Due to dropouts, the data of 120 subjects were analyzed with a mean age of 70.52 years (SD: 8.77 years, range: 50–85 years), whom had been allocated to 4 different groups, each consisting of 30 subjects (SCI, SCI+BEYNEX, AD, AD+BEYNEX). 

The SCI and AD groups’ cognitive performances were analyzed separately, and the SCI and AD groups did not differ significantly in terms of sociodemographic variables or baseline lifestyle activity (Table 1). Among the SCI groups, no meaningful differences were determined between the beginning and the results of the MoCA, GDS, and B-ADL scales (Table 2), whereas the CANTAB results indicated statistically meaningful changes (Table 3). The results of the exercise-practicing group showed positive improvement in parameters of MOT (median), DMS [percent correct and percent correct (all delays)], PAL [total errors (adjusted) and total errors (6 shapes, adjusted)]. The PRM, SWM, and RTI parameters showed no change (Table 3). The AD groups exhibited meaningful differences between the initial and final MoCA, B-ADL, and CANTAB scores. The final MoCA scores of the BEYNEX-practicing group demonstrated a meaningful increase, whereas they decreased in the AD control group. The final B-ADL scores of the BEYNEX-practicing groups meaningfully decreased, indicating an improvement in ADL (Table 2). The CANTAB scores of the BEYNEX groups showed improvement in the parameters of motor screening (MOT), delayed matching sample (DMS), paired associated learning (PAL), pattern recognition memory (PRM), and spatial-working memory (SWM). The reaction time (RTI) parameters showed no change (Table 4). 

**Table 1:** The demographic and lifestyle characteristics within four intervention groups.

Variable	SCI	SCI/BEYNEX	AD	AD/BEYNEX
Age: M (SD)	68.3 (10.94)	66.5 (9.79)	70.85 (7.78)	74.3 (7.56)
Sex: male/female	10/20	13/17	16/14	14/16
Education in years, M (SD)	11.57 (4.42)	13.27(3.66)	11.63 (3.99)	7.83 (3.99)
BMI	29.26 (9.04)	27.46 (4.31)	27.45 (3.21)	28.29 (4.45)
HT	15/30	18/30	19/30	19/30
Exercise	8/30	20/30	21/30	14/30
Smoking	14/30	20/30	16/30	14/30
Alcohol consumption	7/30	10/30	7/30	5/30

Depicted are means (M) and standard deviations (SD) in parentheses. SCI= Subjective cognitive impairment; AD= Alzheimer dementia; BMI= body mass index; HT= hypertension.

**Table 2:** The Cognitive, psychiatric, and activity of daily living assessments.

Variable	Assessment	SCI	SCI/BEYNEX	AD	AD/BEYNEX
MoCA: M (SD)	1st assessment	23.5 (2.91)	24.1 (2.92)	19.07 (2.61)	20.83 (3.39) ***
	2nd assessment	22.03 (5.24)	26.13 (2.87)	16.27 (3.94)	23.23 (3.68) ***
Bayer-ADL (SD)	1st assessment	2.60 (1.46)	2.54 (1.63)	3.54 (1.84)	4.62 (1.89)***
	2nd assessment	2.99 (1.81)	2.42 (1.73)	3.20 (1.66)	3.97 (1.93)***
GDS (SD)	1st assessment	8.33 (4.50)	5.97 (3.31)	5.07 (2.85)	5.57 (3.02)
	2nd assessment	6.70 (1.96)	4.17 (2.77)	3.77 (3.04)	4.70 (2.94)

SCI and AD subgroups were analyzed separately. Depicted are means (M) and standard deviations (SD) in parentheses. Statistical analysis was performed using one-way ANOVA test; ***P < 0.001. SCI= Subjective cognitive impairment; AD= Alzheimer dementia; MoCA= Montreal Cognitive Assessment; B-ADL= Bayer Activities of Daily Living.

**Table 3:** CANTAB test results of SCI groups comparing pre and post CCT (Beynex)

	Mean	Std.Deviation	LowerBound	UpperBound	F	p-Value
MOTmean	836.62	216.88	797.42	875.82	2.45	0.067
MOTMedian	802.99	215.76	763.99	841.99	3.07	0.031*
PRM	78.51	13.35	76.09	80.92	1.07	0.365
DMSA	75.24	14.08	72.70	77.79	3.79	0.012*
DMSB	89.64	14.18	87.08	92.20	2.15	0.097
DMSC	70.45	15.90	67.58	73.33	3.44	0.019
PALA	62.39	54.10	52.61	72.17	4.57	0.005**
PALB	17.58	17.59	14.40	20.75	3.42	0.02*
SWMA	48.63	22.01	44.65	52.60	0.72	0.544
SWMB	34.58	9.88	32.80	36.37	1.06	0.368
SimpleRTIA	8.55	1.08	8.35	8.74	0.36	0.782
SimpleRTIB	405.72	130.98	381.84	429.60	0.90	0.446
SimpleRTIC	374.20	92.47	357.34	391.06	0.89	0.447
SimpleRTID	599.17	182.95	565.81	632.52	1.16	0.328
SimpleRTIE	571.24	158.55	542.33	600.14	1.05	0.373
FiveRTIA	7.87	0.86	7.71	8.02	0.98	0.406
FiveRTIB	435.60	117.91	414.10	457.10	0.44	0.728
FiveRTIC	413.28	98.87	395.25	431.30	0.22	0.881
FiveRTID	565.95	164.02	535.92	595.99	0.74	0.531
FiveRTIE	560.53	146.69	533.55	587.51	0.59	0.625

Statistical analysis was performed using one-way ANOVA test; p<.05 *; p<.01 **

**Table 4:** The CANTAB test results of AD groups comparing pre- and post-CCT results (BEYNEX).

	Mean	Std.deviation	Lowerbound	Upperbound	F	P-value
MOTmean	1130.20	404.63	1057.08	1203.36	6.35	0.001**
MOTMedian	1037.00	360.63	971.78	1102.16	5.73	0.001**
PRM	64.77	16.74	61.75	67.80	10.18	0.001**
DMSA	60.47	17.31	57.34	63.60	7.10	0.001**
DMSB	78.31	22.58	74.22	82.39	9.76	0.001**
DMSC	54.65	18.16	51.37	57.93	4.22	0.01**
PALA	123.64	68.20	111.31	135.97	5.24	0.001**
PALB	35.10	19.03	31.66	38.54	3.39	0.02**
SWMA	54.72	34.99	48.39	61.04	0.98	0.40
SWMB	30.14	15.87	27.27	33.01	6.07	0.001**
SimpleRTIA	8.16	5.38	7.18	9.15	0.28	0.84
SimpleRTIB	525.62	230.18	481.71	569.53	0.76	0.52
SimpleRTIC	476.69	212.91	436.07	517.30	0.46	0.71
SimpleRTID	849.82	377.85	777.40	922.24	0.47	0.70
SimpleRTIE	791.59	373.11	720.08	863.10	0.69	0.56
FiveRTIA	12.19	49.69	2.75	21.62	1.37	0.26
FiveRTIB	545.76	207.39	502.81	588.71	0.88	0.45
FiveRTIC	505.92	192.11	466.13	545.70	0.69	0.56
FiveRTID	714.59	234.79	665.96	763.21	0.38	0.77
FiveRTIE	688.01	202.59	646.05	729.96	0.22	0.88

Statistical analysis was performed using one-way ANOVA test; *P < 0.05; **P < 0.01; ***P < 0.001.

## 4. Discussion

The efficacy of CTT on cognition is a highly debated subject in the medical literature. The cognitive states of individuals and their changes are generally determined by quick scans (e.g., MoCA, MMSE) or other scales. Particularly for individuals with SCI diagnosis, these scales do not signal the change strongly enough. Similarly, our study on MoCA scale CCT showed no changes in the SCI group, whereas in the AD group CCT indicated significant efficacy. Hence, the study included CANTAB to enrich the measurement of cognitive parameters.

In our study, the CANTAB tests for both SCI and AD groups showed significant differences in MOT, DMS, and PAL data. The motor screening test (MOT) is a training procedure designed to relax subjects and to introduce them to the computer and touch screen. It simultaneously screens for difficulties with vision, movement, and comprehension, and it ascertains whether the subject can follow simple instructions, as well as familiarizing them with the touch screen. The DMS is a test of simultaneous and delayed matching-to-sample. This test is primarily sensitive to damage in the medial temporal lobe area, with some input from the frontal lobes. The paired associates learning (PAL) test assesses visual episodic recall memory and new learning, and it is sensitive to medial temporal lobe dysfunction [15]. The spatial working memory (SWM) test is an instrument for assessment of working memory. It is a test of the subject’s ability to retain spatial information and to manipulate remembered items in the working memory. The self-ordered task, which also assesses heuristic strategy, is a sensitive measure of frontal lobe and “executive” dysfunction. The PRM is a test of visual pattern recognition memory in a two-choice forced discrimination paradigm and is sensitive to dysfunction in the medial temporal areas of the brain and relatively insensitive to dysfunction in the frontal lobe. The RTI is designed to measure the subject’s speed of response to a visual target, where the stimulus is either predictable (simple reaction time) or unpredictable (choice reaction time) [15].

The B-ADL has been developed on an international basis to assess deficits in the performance of everyday activities. The scales mainly target patients suffering mild cognitive impairment or mild-to-moderate dementia. It comprises 25 items and takes the form of a questionnaire to be completed by a caregiver or other informant sufficiently familiar with the patient. The scale uses items that reflect a wide range of domains. It is suitable for both screening patients’ ADL capacities and documentation of treatment effects and the progress of dementia [13]. BEYNEX requires users to answer 25 questions once a week about their daily activities. These questions are designed to include activities evaluated by B-ADL. The patients are motivated to be more active by these weekly questions, and the responses (“Yes/No/Not Applicable”) are plotted in a time-series graph for medical professionals to monitor. There are significantly meaningful differences observed on this scale, particularly in patients who are sensitive to cognitive change due to rivastigmine therapy in the AD group. Periodic monitoring of daily activities in an online environment does motivate patients with early-stage AD to adopt a more active lifestyle. The GDS, comprising 30 items, was developed by Yesavage to evaluate depression. Though it showed small changes in the SCI and AD groups, the results are not meaningful.

These findings suggest that CCT produces small improvements in cognitive performance in cognitively healthy older adults but that the efficacy of CCT varies across cognitive domains and is largely determined by the design aspects of CCT. Moreover, because all the included studies measured cognitive function immediately after CCT, these findings provide no information about the durability of the effects of CCT or how the effects of CCT on cognitive function translate into real-life outcomes for individuals such as independence and the long-term risk of dementia. Monitoring the user’s performance over long periods via online software such as BEYNEX can be utilized as a flagging tool for early-stage AD diagnosis, even though it would be difficult to establish a full diagnosis [14].

This study is a rare example of a study that focuses on groups with both SCI and AD. In line with the existing literature, it reiterates that the efficacy of CCT is negligible on SCI patients [16–18]. However, the results also suggest that CCT is more efficient in long-term use (at least 6 months), as well as when it is more accessible online rather than via on-site training. It is important for subjects to integrate CCT into their daily-life routines [19]. In future studies, for systems like BEYNEX, integration with a smart learning algorithm (modest examples of artificial intelligence) may lead to interesting observations in which parameters are more sensitive to change under long-term use of 

CCT in a large number of subjects. There is thus a need for additional research on CCT, an intervention that might help to attenuate age-related cognitive decline and improve the quality of life for older individuals.

In conclusion, this study is a rare example that focuses on both groups with SCI and AD. In line with the existing literature, it reiterates that the efficacy of CCT is negligible in SCI patients but it shows significant efficacy for AD patients. It is important for patients to integrate CCT into their daily-life routines. 

## Acknowledgments 

We sincerely thank Cahit Keskinkılıç (Psychology, Bakırköy Mazhar Osman Hospital, İstanbul, Turkey), Prof. Dr Gülay Kenangil (Department of Neurology, Faculty of Medicine, Bahçeşehir University, İstanbul, Turkey), and Doç. Dr Banu Özen Barut, (Neurology, Lütfi Kırdar Eğitim Araştırma Hospital, İstanbul, Turkey) for their support and willingness to help with our project. 
